# Seven recommendations for scientists, universities, and funders to embrace interdisciplinarity: Practical guidelines to enabling interdisciplinarity

**DOI:** 10.1038/s44319-024-00173-y

**Published:** 2024-06-18

**Authors:** Daniël Paul van Helden, Diane Levine, Eric Guiry, Natalie Darko, Charlotte King, Zahir Hussain, Mukund Janardhanan, Sarah Inskip, Himanshu Kaul

**Affiliations:** 1 School of Archaeology and Ancient History, University of Leicester, Leicester, UK; 2 School of Criminology, Sociology and Social Policy, University of Leicester, Leicester, UK; 3 Centre for Social Development in Africa, University of Johannesburg, Johannesburg, South Africa; 4 The Leicester Institute for Advanced Studies, University of Leicester, Leicester, UK; 5 School of Engineering, University of Leicester, Leicester, UK; 6 Warwick Manufacturing Group, University of Warwick, Coventry, UK; 7 Department of Respiratory Sciences, University of Leicester, Leicester, UK

**Keywords:** Careers, History & Philosophy of Science, Science Policy & Publishing

## Abstract

Successful interdisciplinary research requires proactive efforts by researchers, institutions, funders, and publishers. This article offers practical recommendations at each decision-making level to holistically enhance interdisciplinarity.

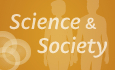

Interdisciplinary research is vital for innovation. Here, we consider interdisciplinarity to mean any form of collaboration between researchers that integrates information, data, techniques, concepts, theories and/or perspectives from two or more disciplines to advance fundamental understanding or solve problems that are beyond the scope of a single discipline (Choi and Pak, 2006; National Academy of Sciences et al, 2005). Increasingly, university leaders, funders and politicians have recognised that the most pressing problems facing the world are too complex to be tackled from a single-disciplinary perspective. Despite this significance and general recognition, a recent report suggests that a high share of academic institutions only pay “lip service” to interdisciplinary research and fail to recognise staff for cross-disciplinary working. Crucially, it states that global research hubs, that is, the USA, UK and Australia, are in reality much less focused on interdisciplinarity versus their Asian counterparts as their research continues to orient itself along disciplinary boundaries and thinking.

… university leaders, funders and politicians have recognised that the most pressing problems facing the world are too complex to be tackled from a single-disciplinary perspective.

While counterintuitive, these conclusions are not surprising given the plethora of practical problems that beset interdisciplinary research. There exists a confusing multitude of labels to indicate different ways in which researchers collaborate between disciplines: interdisciplinary, multidisciplinary and transdisciplinary (see Further Reading, Box 1). The inherent peculiarities mean that interdisciplinary projects take longer to develop, implement and publish, including patents (Lo and Kennedy, 2014) as grasping ideas from other fields is cognitively taxing and time-consuming (Leahey et al, 2017; Vantard et al, 2023); are slower to get cited compared to disciplinary projects (Zhang et al, 2024); have no practical assessment criteria from both funding and publication perspectives (Vantard et al, 2023); enjoy relatively little funding success (Bromham et al, 2016) despite being advertised as funding priority; and can be devalued or outright rejected by disciplinary researchers as interdisciplinary science does not neatly fit into existing schemes (Leahey et al, 2017). This depresses productivity, especially for early-career researchers, with profound implications to their careers (Leahey et al, 2017; Vantard et al, 2023; Zhang et al, 2024). That these factors promote a sense of dissatisfaction (Vantard et al, 2023) amongst practitioners about interdisciplinarity should, therefore, be a cause for concern, not surprise.

Barriers to interdisciplinarity stem from having to fit into an academic landscape that is organised along disciplinary lines (Spanner, 2001). Historically, such an organisation is understandable: as scientists specialised, disciplines developed as the collective fields of interest and, in turn, provided a ‘natural’ organising principle for mechanisms such as funding and employment (van Helden, 2022). Because so much of the academic structure is organised around disciplines, it can be challenging to use academic resources efficiently if one’s research falls between them. This is not limited to funding alone. Research evaluation is also more complex, given that multiple disciplinary standards and criteria need to be reconciled.

The underlying truth is that interdisciplinary collaborations require additional time, a collective willingness to revisit and reflect, patience in stepping into the unfamiliar, and even sacrifices for the greater good.

It is generally accepted that interdisciplinary success relies on the synthesis of disciplinary excellence (Lyall, 2011). The underlying truth is that interdisciplinary collaborations require additional time, a collective willingness to revisit and reflect, patience in stepping into the unfamiliar, and even sacrifices for the greater good. Despite these challenges, the opportunities are plentiful. The following sections highlight some practical challenges encountered in interdisciplinary research and cooperation and offer solutions targeted at individuals, teams, organisations and funders.

## Communication is everything

Arguably, communication-related problems impact all interdisciplinary research teams. Communication with differently trained people, including the uninitiated, is possible, but can fall victim to misunderstandings that are born out of the unwarranted assumption that we all speak the same language, when, in fact, we speak that language in ways that are discipline-specific. For example, industrial engineers may use the word ‘experiment’ to mean simulations; ‘ethical data gathering’ has different connotations for a software developer than for a clinician; a computer modeller may use the word ‘agent’ to describe all ‘discrete’ formulations, but it has a different meaning for software engineers (Kaul and Ventikos, 2015).

This can be overcome via equitable interactions that transcend one-way exchanges of listening and vocalising (see Further Reading, Box 1). Genuinely engage in the communication process to build rapport with your collaborators, and always start with the premise that every team member has something to offer. In the face of competing pressures, it is inevitable that disagreements will occur, sometimes at fundamental levels. This is normal and, if framed in respectful and generous ways, can, in fact, be healthy (Yong et al, 2014).

Collaboration is also easier to maintain when it is based upon a foundation of mutual respect and trust.

Collaboration is also easier to maintain when it is based upon a foundation of mutual respect and trust. Consider the problem from your collaborator’s perspective, with the added benefit that it may, in turn, enable you to suggest novel solutions. This is achieved through engaging with them in conversation or analytic activities to the extent that collaborators try to strengthen each other’s arguments. A good example can be found elsewhere where the authors—biomedical engineers, life scientists, and mathematicians—integrated mathematical formulations with experimental data to capture the molecular logic underpinning developmental patterns (see Further Reading, Box 1). Extreme care was given to using accurate terminology that could not be misinterpreted across disciplines.

Shared approaches to communication, such as non-violent or compassionate communication techniques, can also help to overcome language issues (see Further Reading, Box 1). Such mutual understanding can be facilitated by ‘interdisciplinary translators’ with the explicit role of identifying and overcoming miscommunication (van Helden, 2022). The After the Plague and Arch-I-Scan projects, a collaboration between archaeologists, life scientists and mathematicians respectively, epitomise such approaches to safeguarding translation and communication.

Box 1 Further reading **Definitions of interdisciplinarity**

Wagner CS, et al (2011) Approaches to understanding and measuring interdisciplinary scientific research (IDR): a review of the literature. J Informetrics 5:14–26

Choi BC, Pak AW (2006) Multidisciplinarity, interdisciplinarity and transdisciplinarity in health research, services, education and policy: 1. Definitions, objectives, and evidence of effectiveness. Clin Invest Med 29:351–364

**Communication**

Davidson S (2016) An agnostic critique of the turns to dialogue and symmetry. Public Relations Inquiry 5: 145–167

Gadamer H-G (1990) Warheit und Methode: Grundzüge einer philosophischen Hermeneutik. 6 edn. Mohr Siebeck.

Kaul H, et al (2023) Virtual cells in a virtual microenvironment recapitulate early development-like patterns in human pluripotent stem cell colonies. Stem Cell Rep 18:377–393

Kaul H (2023) Multiscale computational modelling offers key to understanding molecular logic underpinning development and disease. Biotechniques 74:82–285

Museux AC, Dumont S, Careau E, Milot É (2016) Improving interprofessional collaboration: the effect of training in nonviolent communication. Soc Work Health Care 55:427–439

**Common visions and the greater good**

Gavens L, et al (2018) Interdisciplinary working in public health research: a proposed good practice checklist. J Public Health 40:175–182

Blakey ML (2020) On the biodeterministic imagination. Archaeol Dialogues 27:1–16

**Team hierarchy**

Canfield K, et al (2020). Science communication demands a critical approach that centers inclusion, equity, and intersectionality. Front Commun 5:2

Aach J, Lunshof J, Iyer E, Church GM (2017). Addressing the ethical issues raised by synthetic human entities with embryo-like features. Elife 6:e20674

Hurlbut JB, et al (2017) Revisiting the Warnock rule. Nat Biotechnol 35:1029–1042

**Peer review**

McLeish T, Strang V (2016) Evaluating interdisciplinary research: the elephant in the peer reviewers’ room. Palgrave Commun 2:16055

## Establish a common vision

“The only visions that take hold are shared visions”. This is especially true of interdisciplinary research, where visions amongst one set of team members may drastically differ from others, depending on their expertise and background. For example, a 2% improvement in a certain outcome may not be worth pursuing for a mathematician, but a 2% improvement in the number of lives saved can be vital for a clinician. To establish a shared vision, build trust and, consequently, enable teams and individuals to buy into each other’s and the overall vision.

There are numerous ways to achieve this at interpersonal and team levels. Ensure that people value one another rather than seeing interdisciplinary research. This is achieved by identifying shared or reciprocal challenges to overcome by co-creation of methods/tools. Invite a structured conversation, akin to an interdisciplinary ‘prenup’, early on to agree on shared values, visions, objectives, risk appetites, roles and responsibilities, communications protocols, publication strategy, intellectual property, academic impact, risks and opportunities. Notably, this is also an opportunity to proactively address chronic structural inequalities. However, be flexible given that science, and the underpinning relationships, are dynamic, even over short timescales. This approach was used in ‘EVAL-FARMS’, a UK National Environment Research Council-funded project focused on addressing antimicrobial resistance in agriculture that integrated mathematical modelling, engineering, microbial sciences, anthropology and geographical approaches. Having the ‘prenup’ meant that publications, in particular, were managed more equitably across the team.

Such prenups can overcome challenges around authorship, which especially recur in interdisciplinary projects given the requirement for co-creation of ideas/solutions that means individual contributions do not stack against each other in sharp relief. While this is a topic of discussion in its own right, a successful and equitable strategy is to alternate first/last authorships such that those with substantial (and equal) contributions can get due credit.

Last, assigning equal status to all disciplines and being aware of our biases can further reinforce the foundation for the common vision (see Further Reading, Box 1). The Leicester Institute for Advanced Studies, which takes a pluralist approach to trigger collaborative efforts across disciplinary boundaries, is a shining example of mechanisms that universities can exploit to foster interdisciplinary research. Institutes for Advanced Studies championing interdisciplinarity can be found also at Princeton, Stanford, Uppsala, Stellenbosch or University College London.

## Work towards the greater good

Sacrificing disciplinary desirables for the overall goal can elevate the collective outcome beyond a mere juxtaposition of disciplinary results.

Those cooperating across disciplinary borders should be mindful that all parties must offer something in collaboration. And, offer some things up. Sacrificing disciplinary desirables for the overall goal can elevate the collective outcome beyond a mere juxtaposition of disciplinary results. In a collectively written report, integrate approaches and build on each other’s results to enhance the richness of insights generated instead of individual specialist’s reports printed beside one another. Be prepared and expect to be flexible around meeting the most exacting disciplinary standards, since these will not be shared across disciplines, or not to publish exclusively in specific journals, which will force the narrative in a particular direction and sideline other components of the research.

If everyone is prepared to compromise on discipline-specific particularities, a greater synthesis is possible, but the work will also be less prone to catastrophic misrepresentations of collective results. It may also be necessary for individuals to acknowledge difficult positionalities and be prepared to step back from ownership of ideas or activities to address structural inequalities and ensure career-affirming outcomes for the whole.

## Flatten your creative hierarchies

Research innovation is fundamentally a creative endeavour. If research teams are organised in a way that enforces a strict hierarchy—personal or disciplinary—such hierarchies, or the politicking within them, may stifle the creative efforts, especially from those lower down the ladder. In assembling and running research teams, apply EDI (equity, diversity, and inclusion) principles, especially at multiple levels and scales as well as between disciplines and ensure diverse inputs are integrated (see Further Reading, Box 1). For example, make sure during meetings that each team member, regardless of academic positions or discipline, is given an opportunity to contribute. This EDI-focused approach is essential to counter scenarios where more assertive/senior individuals tend to dominate conversations (see Further Reading, Box 1), which would stifle the diversity of perspectives so critical for interdisciplinarity.

If research teams are organised in a way that enforces a strict hierarchy […] such hierarchies, or the politicking within them, may stifle the creative efforts, especially from those lower down the ladder.

Moreover, rotate roles within a project to allow members to experience and understand the challenges and insights of others. For example, a data analyst might take on a stakeholder engagement role, fostering empathy and a deeper appreciation for the communicative and inclusive aspects of research, which in turn can inform more nuanced data analysis. Implement regular EDI monitoring audits to help teams reflect on whether they are truly incorporating diverse perspectives. This reflective practice can lead to the adoption of new research questions that might have been overlooked.

Interdisciplinary training programmes that focus on EDI can help break down silos and build a common language. A biomedical engineering team, for example, could benefit from joint ethics and social science training to ensure that the technologies developed are culturally sensitive and address diverse patient needs. Existing literature on the ethics (see Further Reading, Box 1) of stem cell-based human embryo models and synthetic biology demonstrates such interdisciplinary collaboration between biomedical engineers and ethicists. Flattening the creative process allows insights from junior colleagues or non-specialists, which are potentially less bound by disciplinary constraints, to be voiced and contribute to the discovery of novel solutions.

## Promoting interdisciplinary structures and careers

Achieving interdisciplinarity requires a fertile mindset, which itself is dependent on the research culture. In interdisciplinary institutes, the nuanced ‘cultural’ differences between disciplines can result in real, problematic differences in understanding, research practice and delivery, especially where they go unrecognised (van Helden, 2022). While shaping an interdisciplinary research culture is non-trivial, universities/institutes can support this by creating mechanisms dedicated to bringing people together to both identify new synergies and maintain the momentum to harness them.

This includes hiring practices that promote the integration of diverse teams with complementary expertise and creating institutes or centres that do not segregate distinct disciplines or reinforce structural inequalities in higher education. Set up a robust governance structure that includes experienced interdisciplinarians from beyond the institution. The entity should not sit in the administration department of the university but should be understood and financially supported as a home across all faculties. Ensure the entity has someone with project/programme leadership experience to support effective decision-making.

Furthermore, the importance of fresh, external perspectives such as those exemplified by Visiting Fellowship schemes are crucial to counteracting groupthink. Such endeavours can give rise to new teams, fresh ideas and profound perspectives that foster and beget similar thinking and activities. This can be further supported by providing appropriate training and resources required to promote interdisciplinarity, including supporting a ‘locus of interdisciplinarity’ that serves the needs of all collaborators (Lyall et al, 2011).

## Train interdisciplinary peer reviewers

The inability to find suitable reviewers is a common refrain from academic journals (see Further Reading, Box 1), even those dedicated to publishing interdisciplinary research. This comes at a huge cost to interdisciplinary researchers who either get their manuscripts rejected because of sub-optimal reviews or may have to wait significantly longer for peer review and, thus, publication (Leahey et al, 2017; Lo and Kennedy, 2014). While pre-prints have helped to balance this issue to some degree, it slows down scientific progress with negative implications on the careers of interdisciplinary researchers (Leahey et al, 2017; Vantard et al, 2023; Zhang et al, 2024).

This applies equally to funding applications that get unfairly rejected due to a lack of suitable reviewers. This issue can be overcome by assigning reviewers—to both grant proposals and manuscripts—with a track record of conducting/leading interdisciplinary research. As this is not always possible, editors and grant managers can instruct disciplinary reviewers with specific criteria, encourage them to think outside the box, keep an open mind to extra-disciplinary concepts, and consider the wider view and impact of the article under review. Discipline-specific reviewers’ expertise will, of course, be relevant in scenarios where interdisciplinary research ends up challenging what may be considered dogma in a field, however, prominence must be given to the comments/decision of the interdisciplinary reviewers with specific caveats introduced in the publication.

Importantly, reviewers and editors can work together to integrate the views of different disciplinary reviewers in a constructive way. In comparison, manuscripts/proposals where a particular tool or method from one field is applied in a different field with no reciprocal benefit—such as using an AI model to develop gene regulatory network models—should be sent to subject-specific reviewers. To enable rapid review, journals and funders should have access to interdisciplinary peer reviewers, not just on the editorial board, with a broader research interest who can offer quick feedback. Publishers should also assist reviewers with specific guidelines to assess interdisciplinary manuscripts. The UKRI Interdisciplinary Assessment College is at the forefront of achieving this step change.

## Funding interdisciplinary research and personnel is key

Another step that universities and funders can take is to promote studentships/scholarships to specifically encourage interdisciplinary collaborations and promote interdepartmental doctoral supervision. The BBSRC-funded Midlands Integrative Biosciences Training Partnership and MRC-funded Advanced Interdisciplinary Models studentships represent salient examples of such mechanisms. Such training opportunities will go some way towards providing the requisite interdisciplinary reviewers discussed above.

Further, funding bodies can have periodic calls specifically to attract and fund new interdisciplinary teams and ideas. The success of such dedicated calls needs to be monitored, as the tension between appetite for novelty and expense on the part of the funder is not always aligned with similar levels of appetite from reviewers (Marsden et al, 2011). There is a body of evidence supporting research funders in commissioning interdisciplinary research (Marsden et al, 2011; Strang and McLeish, 2015), and signs that the funding landscape is slowly moving in the right direction. UK Research Council’s recent cross-Council calls focusing on intersections between health, and the natural and heritage/historic environments are welcome examples, as are the European Research Council’s Pathfinder calls. Additionally, the Leverhulme Trust has placed interdisciplinarity at the heart of its commissioning process for many years, as have the UK’s national academies. While these remain a fraction of the overall funding landscape, they still represent a cause of hope. Importantly, however, fixed-term funding is only ever going to solve so much.

Unless interdisciplinary researchers/technicians have options for permanent employment and, through that, the opportunity to contribute long-term to research cultures, all measures discussed here will be limited stop-gap fixes. Interdisciplinarity will never become truly viable as a career path if its practitioners are doomed to precarious temporary employment. Therefore, when hiring or reviewing promotion candidates for permanent positions, do not penalise interdisciplinary researchers by judging them with the same yardstick—typically the number of papers published and grants secured—as their (multi)disciplinary peers, given that interdisciplinary science takes more time to be implemented, funded, conducted and published. This is best evidenced by a recent survey (Vantard et al, 2023) where a mere 27% of interdisciplinary respondents felt that their research has had a positive impact on their career. This requires a fundamental rethink at the institutional level and active engagement with the research community to develop relevant criteria for measuring interdisciplinary careers. As highlighted by Vantard et al (2023), the European Coalition for the Advanced Research Assessment’s (CoARA’s) five major principles of *quality, impact, diversity, inclusiveness, and collaboration* as the underpinning criteria to assess interdisciplinary careers—and indeed projects and organisation—allows for more comprehensive evaluation.

Interdisciplinarity will never become truly viable as a career path if its practitioners are doomed to precarious temporary employment.

## Conclusions

Interdisciplinarity depends on navigating a pluralistic lens, which requires curiosity and an acknowledgement that the process is neither linear nor achievable without collective input. Here we suggest improvements that can be made by facilitating the establishment of new communication channels through institutional support, mitigating potential misunderstandings due to differences in disciplinary culture, encouraging creative conversations and not stifling them with entrenched expectations or hierarchies, developing relevant criteria for measuring interdisciplinary careers, and adopting an approach underpinned by EDI policies. Choosing the roads less travelled will always involve having to overcome practical obstacles not encountered by those who do not cross disciplinary boundaries. While not ever straightforward, this path is almost always rewarding though.

### Supplementary information

Peer Review File
