# Supplementary material for: Seven recommendations for scientists, universities, and funders to embrace interdisciplinarity: Practical guidelines to enabling interdisciplinarity
Source: EMBO Rep. 2024 Jun 18;25(7):2832–6. doi: 10.1038/s44319-024-00173-y (PMC11239911; doi:10.1038/s44319-024-00173-y)
Supplement: Supplementary file 1 — Peer Review File [file 44319_2024_173_MOESM1_ESM.pdf]

EMBO reports

Practical guidelines to enabling interdisciplinarity

Daniël van Helden, Diane Levine, Eric Guiry, Natalie Darko, Charlotte King, Zahir Hussain, Mukund Janardhanan, Sarah Inskip, and Himanshu Kaul

Corresponding author(s): Himanshu Kaul (himanshu.kaul@leicester.ac.uk)

Review Timeline:

Submission Date:	10th May 24
Accepted:	3rd Jun 24

Editor: Holger Breithaupt

Transaction Report: This article was editorially reviewed.
